# Perceptions, Challenges, and Operational Bottlenecks in Electronic Vaccine Intelligence Network Implementation: A Qualitative Study Among Immunization Stakeholders in Indore District, India

**DOI:** 10.7759/cureus.112254

**Published:** 2026-07-08

**Authors:** Sana Afrin, Dolly Mantri, Shivam Dixit, Abhishek Singh

**Affiliations:** 1 Department of Community Medicine, Sri Aurobindo Medical College and Post Graduate Institute, Indore, IND; 2 Department of Community Medicine, Mahatma Gandhi Memorial Medical College, Indore, IND; 3 Department of Community Medicine, Shaheed Hasan Khan Mewati (SHKM) Government Medical College, Nuh, IND

**Keywords:** cold-chain management, digital health, evin, immunization supply chain, vaccine logistics

## Abstract

Background: National assessments of the Electronic Vaccine Intelligence Network (eVIN) have demonstrated reductions in vaccine stock-outs and wastage, improved cold-chain equipment performance, and strengthened data-driven stock management. However, few studies have qualitatively explored stakeholders' perspectives on its routine implementation, perceived benefits, and operational challenges.

Objective: This study aimed to explore immunization stakeholders' perceptions of the benefits, challenges, and operational bottlenecks of the eVIN in Indore district and to identify system-level improvements for strengthening vaccine logistics and cold-chain performance.

Methods: This descriptive qualitative study was conducted in Indore district, Madhya Pradesh, India, between January 2020 and December 2021. Using total population sampling, all key eVIN stakeholders associated with 40 cold-chain points (CCPs) were invited to participate, including district-level officials and facility-level staff. Data were collected through face-to-face semi-structured interviews, transcribed verbatim, and analyzed using reflexive thematic analysis with investigator triangulation.

Results: Stakeholders perceived improvements in real-time stock visibility, distribution efficiency, continuous temperature monitoring, reduced vaccine wastage, and standardized record-keeping following eVIN implementation. Participants also identified important limitations, including dependence on accurate data entry, the lack of beneficiary-level immunization data, human resource shortages, unreliable Internet connectivity, device malfunctions, and duplication of paper and electronic records.

Conclusions: Stakeholders perceived that eVIN strengthened vaccine logistics and cold-chain monitoring in Indore district. However, persistent challenges related to workforce capacity, infrastructure, funding, and system integration continue to limit its optimal use. Addressing these gaps may improve the sustainability and effectiveness of eVIN in routine immunization services.

## Introduction

Globally, immunization supply chains are recognized as a critical backbone of national immunization programs, and persistent weaknesses in cold-chain equipment, real-time stock visibility, and temperature monitoring continue to limit coverage gains, particularly in low- and middle-income countries. Recent WHO guidance emphasizes end-to-end visibility of vaccine flows, robust temperature monitoring, and data-driven stock management as core pillars of resilient immunization supply chains [1]. Digital information systems, including electronic stock management tools and dashboard-based monitoring, have therefore been promoted to address long-standing challenges such as stock-outs, vaccine wastage, and fragmented data.

India’s Universal Immunization Programme is one of the largest in the world, protecting millions of children and pregnant women against vaccine-preventable diseases. An efficient vaccine supply chain is essential to ensure that vaccines remain available and potent until the point of last-mile delivery [2-4]. However, despite the success of the routine immunization program, the national vaccine supply chain continues to face operational challenges.

In 2015, the Ministry of Health and Family Welfare, with support from the United Nations Development Programme (UNDP), introduced the Electronic Vaccine Intelligence Network (eVIN) in selected states to modernize immunization logistics [5,6]. eVIN is a smartphone- and cloud-based application that digitizes information on vaccine stock levels and cold-chain temperatures at all vaccine storage points, enabling real-time visibility for program managers [6]. Over the subsequent years, eVIN was scaled up to cover all 731 districts across 36 states and union territories of India [7].

Previous assessments of eVIN in India have primarily focused on quantitative indicators such as stock-out rates, vaccine utilization trends, and cold-chain equipment performance, demonstrating substantial improvements following its implementation [6,7]. However, there is relatively limited district-level qualitative evidence on how stakeholders experience eVIN in routine practice. Understanding stakeholders' perspectives is essential for identifying practical challenges and barriers that may not be apparent through quantitative data alone. Therefore, the present study was conducted to qualitatively explore the perceptions of immunization stakeholders in Indore regarding the implementation of eVIN.

## Materials and methods

The present descriptive qualitative study was conducted in Indore district, Madhya Pradesh, India, between January 2020 and December 2021. Institutional Ethics Committee approval was obtained before the initiation of the study (Letter No. EC/MGM/Dec-19/16), and written informed consent was obtained from each participant.

A total population (census) sampling approach was used to invite all eligible stakeholders involved in the eVIN across the 40 cold-chain points (CCPs) in Indore district, including district-level officials and facility-level staff. This approach was chosen because the number of individuals in each stakeholder cadre was small, and all were considered critical informants for eVIN implementation. Complete inclusion, rather than purposive sampling, ensured representation of the distinct perspectives of each stakeholder group (e.g., chief medical and health officer (CMHO), district immunization officer (DIO), vaccine and cold-chain manager (VCCM), district vaccine store manager (DVSM), cold-chain technician (CCT), medical officers (MOs), and cold-chain handlers (CCHs)). Recruitment was attempted for all eligible individuals, and interviews continued until thematic saturation was achieved across stakeholder cadres. Interviews were conducted with all consenting participants until no new substantive codes emerged across successive transcripts within each cadre (code saturation), and the existing themes were sufficiently developed, with no new conceptual dimensions emerging (meaning saturation). Saturation was jointly assessed by the two primary coders in consultation with a senior qualitative researcher, following established qualitative research guidance that distinguishes between code and meaning saturation (Table 1).

**Table 1:** Stakeholder allocation matrix for Electronic Vaccine Intelligence Network (eVIN) implementation in Indore district Values are presented as the number of stakeholders (n, % of total N = 85).

Stakeholder cadre	Level	Primary role in eVIN implementation	n (%)
Chief medical and health officer (CMHO)	District	Overall leadership and oversight of the Universal Immunization Programme and eVIN implementation	1 (1.2)
District immunization officer (DIO)	District	Program management, supervision of immunization activities, and use of eVIN dashboards for decision-making	1 (1.2)
Vaccine and cold-chain manager (VCCM)	District	Coordination of eVIN roll-out, training and supportive supervision of cold-chain handlers, and routine data quality checks	1 (1.2)
District vaccine store manager (DVSM)	District	Management of the district vaccine store, stock planning, and distribution to cold-chain points using eVIN data	1 (1.2)
Cold-chain technician (CCT)	District	Installation, preventive maintenance, and repair of cold-chain equipment and temperature loggers	1 (1.2)
Medical officers (MOs)	Facility	Facility-level supervision of immunization sessions, oversight of vaccine and cold-chain equipment management, and use of eVIN reports	40 (47.1)
Cold-chain handlers (CCHs)	Facility	Day-to-day vaccine stock management, temperature monitoring, and data entry into the eVIN application at cold-chain points	40 (47.1)

Sample size in this descriptive qualitative study was determined by inclusion of the complete universe of eligible stakeholders and confirmation of thematic saturation rather than by an a priori statistical power calculation. Written informed consent was obtained from each participant, including permission to audio-record the interview. Participant confidentiality was ensured throughout the study. An interview guide was used to ensure consistency across interviews while allowing flexibility to probe for additional details. Data were collected through face-to-face, semi-structured interviews conducted at participants' workplaces. Each interview lasted approximately 30-45 minutes. Because data collection overlapped with the COVID-19 pandemic, interviews were conducted in accordance with institutional and government infection-prevention guidelines, including mask use, physical distancing, and adequate ventilation during face-to-face interviews. The interviews were conducted in Hindi, and the responses were translated into English during transcription.

The interviews were conducted by two public health researchers with training in qualitative methods and prior experience working with immunization programs who were not directly responsible for eVIN implementation or supervision in Indore. The interviewers introduced themselves as independent researchers rather than program supervisors. Interviews were conducted in private settings to reduce perceived pressure, and participants were assured that their responses would remain confidential and would not affect their employment or performance evaluations.

The semi-structured interview guide was developed using national and global guidance on immunization supply chains and eVIN implementation and was informed by the Ministry of Health and Family Welfare's Techno-Economic Assessment of eVIN and related evaluation reports. The guide was reviewed by three senior public health experts to establish content validity and was piloted with two eVIN stakeholders outside the study district. No standardized psychometric scales were used, and no additional instrument licenses were required.

**Figure 1:**
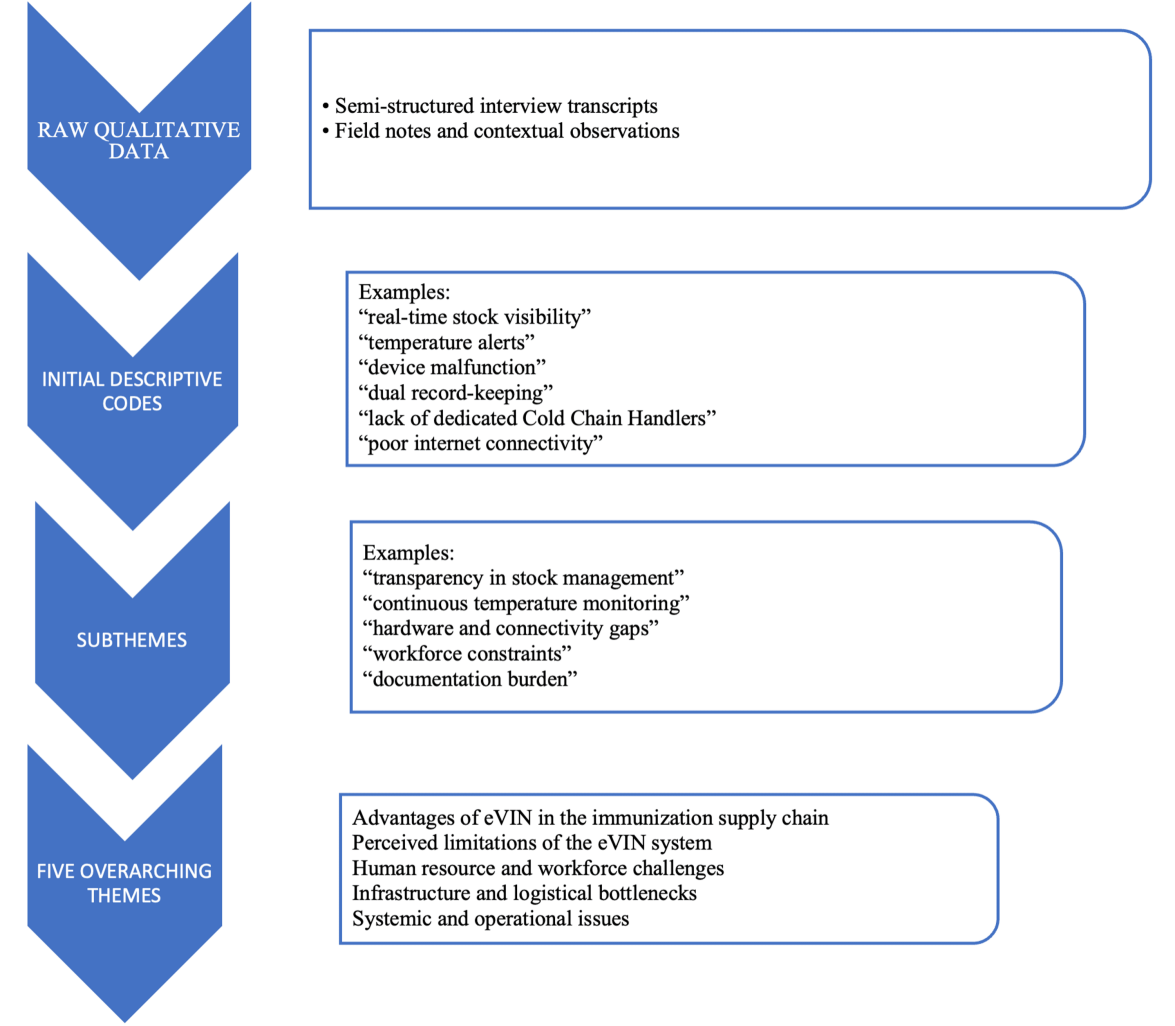
Thematic coding framework for eVIN implementation in Indore district eVIN: Electronic Vaccine Intelligence Network. Note: This figure was created using Microsoft PowerPoint 2026 (Microsoft Corporation, Redmond, WA, USA).

In addition to the audio recordings, brief field notes were taken. All interviews were transcribed verbatim and translated into English, and the transcripts were cross-checked for accuracy to preserve the original meaning. As this study adopted a descriptive qualitative design, the data were analyzed using reflexive thematic analysis following the six-phase framework described by Braun and Clarke (2006) [8]. The thematic coding framework is presented in Figure 1.

Two investigators independently coded all transcripts, met regularly to reconcile their coding frameworks, and developed a shared codebook. Codes were then grouped into subthemes and overarching themes through an iterative process of discussion, with discrepancies resolved through consensus and, when necessary, adjudication by a senior qualitative researcher. Data were organized and managed using Microsoft Excel, version 2026 (Microsoft Corporation, Redmond, WA, USA), and coding was performed in Microsoft Word, version 2026. No dedicated qualitative data analysis software was used.

## Results

A total of 81 initial codes were consolidated into 16 categories, which were then further refined into 5 overarching themes that structure the findings. Theme development followed the reflexive thematic analysis framework outlined by Braun and Clarke [8], with repeated movement between the data, codes, and candidate themes.

The findings are presented under the following themes.

Theme 1: advantages of eVIN in the immunization supply chain

Real-Time Stock Visibility

The CMHO, DIO, VCCM, and DVSM highlighted the ability to view vaccine stock levels in real time across all CCPs in the district. This feature enables rapid access to inventory information. As one MO stated, “With eVIN, I can instantly check how many doses are available at my facility at any time.” District officials noted that this transparency extends throughout the supply chain, allowing them to identify potential shortages or overstocks at a glance. According to the DIO, “The vaccine stock and flow across all our facilities can be viewed in real time, which helps a lot in decision-making.” This real-time overview has made it easier to maintain adequate stock levels at every CCP and prevent stock-outs.

Streamlined Distribution and Stock Management

Respondents reported that vaccine distribution from the district store to peripheral facilities has become more systematic. As the DVSM stated, “eVIN has helped us plan distribution better and ensure each point gets the right quantity.” Automatic calculation of vaccine requirements, based on preset minimum stock levels and consumption, was highlighted by a CCH as a useful feature that generates timely indents. As a result, stock-outs have become infrequent, and redistribution of surplus stock between facilities is better coordinated. Several participants credited eVIN with virtually eliminating facility-level vaccine shortages in Indore. As one MO noted, “An adequate level of vaccine stock is maintained at our CCP most of the time.”

Improved Cold-Chain Temperature Monitoring

All stakeholders appreciated eVIN's contribution to the continuous temperature monitoring of ice-lined refrigerators (ILRs). eVIN introduced digital temperature loggers that automatically record and transmit storage temperatures. As a CCH explained, “Earlier, we recorded temperatures only twice a day on paper. Now, the temperatures are also tracked 24/7 through the temperature loggers, and we receive alerts if there is any breach.” The real-time temperature data, accessible through the eVIN app and dashboard, allows timely corrective action if a refrigerator goes outside the recommended temperature range. As the CCT noted, “With eVIN, any temperature breach is immediately noticed and acted upon, ensuring vaccine potency is maintained.” The CMHO also stated, “The temperature of the cold chain equipment at all CCPs can be viewed in real time whenever required.” 

Reduced Vaccine Wastage

Several participants reported that improved stock management and temperature monitoring have resulted in reduced vaccine wastage. By preventing overstocking and vaccine expiry through first-expiry-first-out (FEFO) inventory management, and by minimizing incidents of temperature excursions, eVIN has helped preserve vaccine doses. As one MO stated, “Vaccine wastage has significantly reduced. With eVIN, we plan stock more accurately and monitor temperatures closely, so wastage has come down.”

Standardized and Accountable Record-Keeping

The introduction of eVIN was accompanied by standardized registers and protocols for recording vaccine stock and temperature at each CCP. CCHs noted that eVIN provided uniform vaccine stock registers and temperature logbooks, replacing diverse and, at times, inconsistent local record-keeping formats. In addition, maintaining digital records in the eVIN application provides an audit trail, thereby increasing accountability. As the VCCM remarked, “Documentation is now more complete and accurate. The data recorded in eVIN can always be cross-checked, which wasn't possible with scattered paper records.” 

Theme 2: perceived limitations of the eVIN system

Dependence on Data Accuracy

District officials pointed out that if incorrect or incomplete data are entered into the eVIN app by the CCH, the system will propagate those errors to everyone viewing the dashboard. As the DIO explained, “If the CCH makes a wrong entry, eVIN will show incorrect stock information. Officials might develop a false sense of security or panic unnecessarily.” Thus, eVIN does not inherently prevent human errors at the point of data entry, and poor data quality can undermine its effectiveness. 

Lack of Immunization Coverage Data in eVIN

A commonly mentioned limitation was that the eVIN application focuses solely on vaccine logistics, including vaccine stock management and cold-chain monitoring, and does not capture information on the number of beneficiaries vaccinated. As the CMHO noted, “eVIN tells us about the vaccines, but not about the vaccination. It doesn’t show how many beneficiaries received the vaccine.” Stakeholders felt that if eVIN could also display the number of doses administered, it would provide a more comprehensive picture and help correlate vaccine supply with utilization. 

Theme 3: human resource and workforce challenges

Lack of Dedicated CCHs

One systemic bottleneck identified was the absence of full-time dedicated CCHs at the study facilities. The role of the CCH was often assigned as an additional responsibility to multipurpose health workers, nurses, or pharmacists. As one MO explained, “We don’t have staff whose only job is managing the cold chain. It’s always someone doing it on top of their regular duties.” Participants noted that this arrangement can reduce the attention devoted to eVIN-related tasks. Stakeholders across all levels emphasized the need to appoint full-time CCHs at every CCP who are dedicated exclusively to cold-chain management, thereby improving focus and accountability for eVIN implementation and cold-chain maintenance.

Shortage of Backup Personnel (Alternate CCHs)

Consistent with the above findings, another challenge was the lack of trained backup cold-chain staff to cover the responsibilities of the primary CCH during periods of absence. Many respondents reported that “If our CCH is on leave or unavailable, there’s no one else who knows the system well,” leading to interruptions in eVIN updates and suboptimal vaccine management. Several facilities also lacked a formally designated backup or “link person” to serve as an alternate for the CCH.

Need for Continuous Training and Retraining

Training was universally acknowledged as essential for the effective implementation of eVIN. All participants had undergone initial eVIN training and refresher sessions. However, higher-level stakeholders (CMHO, DIO, and VCCM) identified the need for repeated training whenever the eVIN application was updated or new features were introduced as an ongoing challenge. They observed that frequent application updates required additional refresher training for staff, which was time-consuming and sometimes difficult to organize regularly. As the VCCM stated, “Every time there’s an update, we have to re-train everyone so they know the new process. It’s hard to keep up.” Additionally, staff transfers and turnover within the government health system meant that newly appointed personnel often had not received eVIN training, creating an ongoing need for additional training. In contrast, frontline CCHs did not identify training as a major concern, possibly because they had already benefited from the available training. From a management perspective, however, maintaining a consistently well-trained workforce was regarded as a continuing challenge.

Theme 4: infrastructure and logistical bottlenecks

Inadequate Internet Connectivity

The most frequently reported challenge was unreliable internet or cellular network connectivity at certain CCPs. CCHs from various sites described difficulties uploading stock data through the eVIN application when network connectivity was poor. As one rural CCH commented, “Sometimes the network is so slow or down that I keep trying for hours to upload the data.” Poor connectivity delayed the timely updating of information in the system and often frustrated users. As a result, some CCHs entered data at night from home when network connectivity was better, while others moved to areas within the facility where the signal was stronger.

Mobile Device Issues

The implementation of eVIN included the provision of smartphones for CCHs to operate the eVIN application. However, multiple stakeholders reported that the smartphones provided frequently malfunctioned or were of poor quality. As one CCH reported, “The phone they gave us hangs a lot, and the battery drains quickly.” In several cases, the devices became non-functional within one to two years. As a result, many CCHs resorted to using their personal smartphones to operate the eVIN application, raising concerns about personal costs and device compatibility. Participants also noted that there was no dedicated district-level funding for procuring replacement smartphones when the existing devices failed.

Temperature Logger Functionality

In addition to smartphones, stakeholders reported that the SIM-enabled digital temperature loggers installed in ILRs and freezers were occasionally non-functional. When a logger stopped working because of a technical failure or SIM/network issues, continuous temperature monitoring was interrupted. As one CCH stated, “The logger in one of our fridges hasn’t sent data for weeks, so we have to rely on manual readings again.” Participants emphasized the need for timely maintenance or replacement of faulty temperature loggers to ensure uninterrupted monitoring.

Insufficient Cold-Chain Logistics Funding

A critical bottleneck identified by many participants was the lack of dedicated funding for cold-chain maintenance and operations at the facility level. Cold-chain-related expenses were often covered through the NHM flexi-pool, without a dedicated budget line. As a result, CCP staff sometimes paid out of pocket or relied on ad hoc measures to meet operational needs. For example, urgent vaccine deliveries outside the regular schedule were challenging because of the lack of dedicated transportation, as most CCPs did not have access to vehicles beyond the periodic alternate vaccine delivery system.

Supply of Consumables (Log Books and Registers)

Related to funding, CCHs pointed out that the standardized paper registers and temperature logbooks provided under eVIN occasionally ran out and were not replenished in a timely manner. As one CCH described, “When our logbook was filled, we didn’t receive a new one for months, so we photocopied pages to use in the meantime.” Participants emphasized that ensuring a continuous supply of these materials is a seemingly minor but important administrative requirement for the effective functioning of eVIN.

Theme 5: systemic and operational issues

Duplication of Record-Keeping

A frequently mentioned concern among CCHs was the burden of duplicate data entry. Even after the introduction of eVIN, the requirement to maintain both electronic records in the eVIN application and physical paper registers persisted. As one CCH stated, “We have to do everything twice. It definitely takes more time.” Participants noted that this duplication was tedious and increased their workload. One MO suggested that complete digitization at the point of service would simplify the process and reduce the documentation burden.

User Interface and App Updates

Some CCHs mentioned that the order of vaccine names in the app’s dropdown menus did not match the order in the paper register, causing potential confusion during entry. “In the register, vaccines are listed in one order, but in the app it’s different, so initially I would accidentally select the wrong one,” one CCH recalled. More significantly, the frequent updates to the eVIN app were cited as an operational challenge. Participants noted that while improvements are welcome, updates often came without sufficient notice or clear documentation in the local language, leaving users to adapt on their own until formal training caught up. District officials supported this, saying they needed to conduct refreshers after major updates to prevent user errors.

## Discussion

The present study indicates that stakeholders perceived that eVIN strengthened vaccine stock management and cold-chain monitoring at both the district and facility levels. Participants commonly described improvements such as real-time stock visibility, streamlined distribution, and enhanced temperature monitoring, which are consistent with the intended objectives of eVIN. These qualitative perceptions are consistent with the quantitative evaluation by Gurnani et al. [9], which documented significant reductions in vaccine wastage and stock-outs.

The Techno-Economic Assessment of eVIN also reported similar benefits, including improved stock management practices, streamlined vaccine distribution through real-time visibility of vaccine stocks across all CCPs, reduced stock-outs and vaccine wastage, and better management of cold-chain equipment through trained CCHs [5]. Furthermore, cold-chain equipment, which received comparatively less attention before eVIN implementation, assumed greater importance following its introduction [10]. These findings are supported by programmatic reports as well as independent evaluations demonstrating improvements in stock management, temperature monitoring, and logistics efficiency.

The ability to monitor cold-chain equipment temperatures in real time through digital temperature loggers enabled timely corrective action in the event of temperature breaches. Similar findings were reported by Chaudhary et al. [7], who described improved capacity building through staff training and deployment of VCCMs, strengthened planning and decision-making using real-time dashboards, and enhanced temperature monitoring through digital loggers. Participants in their study also highlighted reductions in vaccine wastage, improved assurance of vaccine potency, and the need for sustained investment in infrastructure, human resources, and equipment maintenance.

The success of eVIN in India has also attracted international attention. Countries such as Indonesia have adopted India's eVIN technology for their immunization programs, while UNDP has supported the implementation of similar systems in several African and Asian countries [11]. These experiences suggest that the findings from Indore reflect broader evidence supporting digital vaccine logistics systems for strengthening immunization supply chains.

Despite these perceived benefits, participants consistently identified human resource limitations as a major challenge. None of the CCPs had a full-time CCH dedicated exclusively to cold-chain management. Instead, these responsibilities were assigned as additional duties to multipurpose health workers, nurses, pharmacists, or other staff. Several facilities also lacked trained backup CCHs to manage cold-chain operations during staff absences. Similar observations were reported by Lohani [12], who noted that existing personnel were assigned CCH responsibilities rather than appointing dedicated staff. Likewise, the Techno-Economic Assessment of eVIN recommended appointing full-time CCHs and additional backup personnel trained specifically for eVIN implementation [5]. These findings are consistent with broader evidence from Rao et al. [13], who reported substantial shortages of doctors, nurses, and midwives in India, affecting the delivery of essential public health services, including immunization. Frequent staff transfers and the resulting need for repeated training further compound these workforce challenges [8].

Participants also highlighted several infrastructure-related barriers that limited the optimal use of eVIN. Unreliable Internet connectivity, malfunctioning smartphones, and non-functional SIM-enabled temperature loggers frequently disrupted real-time data reporting and temperature monitoring. Similar challenges have been reported by Chaudhary et al. [7], particularly in remote and rural health facilities, while Montgomery et al. [14] emphasized the importance of reliable network connectivity for maintaining real-time information systems. Participants also reported shortages of temperature logbooks at CCPs with multiple ILRs or deep freezers, reflecting broader issues related to maintenance, logistics, and funding. Similar operational gaps, including malfunctioning smartphones, inadequate stationery supplies, network issues, insufficient replacement policies for equipment, and funding constraints, were identified in the Techno-Economic Assessment of eVIN [5]. More broadly, comparable challenges have also been described in evaluations of digital immunization supply chain systems, where limited connectivity, inadequate equipment maintenance, and weak replacement mechanisms reduced system effectiveness. These findings suggest that sustained investment in connectivity, equipment maintenance, reliable power supply, and dedicated funding mechanisms is essential for ensuring the long-term sustainability of digital vaccine logistics systems.

Another important operational issue identified in the present study was the continued requirement to maintain parallel paper and electronic records, resulting in duplicate documentation and increased workload. Similar concerns were highlighted in the Techno-Economic Assessment of eVIN [5]. Participants also reported difficulties adapting to frequent application updates, particularly when updates were introduced without adequate user guidance. These findings suggest that future system updates should incorporate user feedback, provide clear documentation in local languages, and include concise user guides or in-app tutorials. Participants also expressed concern that eVIN does not capture beneficiary-level vaccination data. Integrating eVIN with other health information systems, such as electronic immunization registries or the CoWIN platform, could enable linkage of vaccine supply and service delivery data, thereby providing program managers with a more comprehensive overview of immunization performance, as suggested in other digital health studies [15].

Beyond workforce-related constraints, participants' accounts of unreliable Internet connectivity, malfunctioning smartphones, and non-functional temperature loggers underscore the central role of infrastructure and hardware maintenance in sustaining digital health interventions. Similar challenges have been documented in other evaluations of eVIN and immunization supply chain information systems, where weak connectivity, limited device life cycles, and underfunded repair and replacement mechanisms have undermined otherwise well-designed digital platforms [16]. These observations suggest that investments in reliable connectivity, power backup, and clear protocols and dedicated budgets for device and equipment maintenance are not merely supportive but are essential for realizing the full benefits of real-time, data-driven supply chain management [17].

Limitations

This study has several limitations that should be considered when interpreting the findings. First, social desirability bias may have influenced participants' responses, as some may have emphasized program strengths while discussing eVIN implementation. Second, although interviewer bias was minimized through interviewer training, the use of a semi-structured interview guide, and reflexive discussions during data analysis, it cannot be completely excluded. Third, because the interviews were conducted in Hindi and subsequently transcribed and translated into English, some translation bias may have occurred despite careful cross-checking for accuracy. Fourth, recall bias is possible, particularly in participants' retrospective accounts of pre-eVIN conditions. Finally, the study was conducted in a single, predominantly urban district with comparatively better infrastructure, which may limit the transferability of the findings to rural or more resource-constrained settings. However, a thick description of the study context has been provided to enable readers to assess the applicability of the findings to other settings.

To further protect participant confidentiality, direct quotations were attributed using broader role descriptors rather than specific local positions, and all participants provided informed consent for the use of de-identified quotations.

## Conclusions

In this study, stakeholders perceived that eVIN strengthened vaccine logistics and cold-chain monitoring in Indore district. Participants reported improvements in stock visibility, distribution planning, temperature monitoring, and record-keeping. However, they also identified persistent challenges related to human resources, infrastructure, funding, and system integration. Addressing these gaps through dedicated workforce support, improved connectivity, regular device maintenance, and better integration with other health information systems may enhance the sustainability and effectiveness of eVIN in routine immunization services.
